# Dry-Cured Sausages “Salchichón” Manufactured with a Valorized Ingredient from Red Grape Pomace (Var. Tempranillo)

**DOI:** 10.3390/foods13193133

**Published:** 2024-09-30

**Authors:** Matilde D’Arrigo, María Jesús Petrón, Jonathan Delgado-Adámez, Jesús Javier García-Parra, María Jesús Martín-Mateos, María Rosario Ramírez-Bernabé

**Affiliations:** 1 Centro de Investigaciones Científicas y Tecnológicas de Extremadura (CICYTEX), Instituto Tecnológico Agroalimentario (INTAEX), 06007 Badajoz, Spain; matilde.darrigo@juntaex.es (M.D.); jonathan.delgado@juntaex.es (J.D.-A.); jesusjavier.garcia@juntaex.es (J.J.G.-P.); mariajesus.martinmat@juntaex.es (M.J.M.-M.); 2 Escuela Ingenierías Agrarias, Universidad de Extremadura, 06006 Badajoz, Spain

**Keywords:** dry-cured sausages, red grape pomace, phenolic compounds, antioxidant, nitrifying salts

## Abstract

The inclusion of an ingredient made from red grape pomace (RGP) var. Tempranillo was evaluated for the preservation of a traditional dry-cured sausages (salchichón). The pomace was valorized through thermal blanching (103 °C for 1 min) and hydrostatic high-pressure treatment (600 MPa/5 min) before the addition to salchichón. Four formulations of salchichón were evaluated, including a negative control (NC—without red grape pomace or synthetic additives), positive control (PC—with ascorbic acid and nitrites), low level (LL—0.5%), and high level (HL—1%) of RGP. Physicochemical, microbiological, and sensorial effects were analyzed. RGP reduced the final pH of salchichón and favored the growth of lactic acid bacteria at similar levels as PC. The addition of ascorbic acid and nitrites resulted in a final product with a redder and less yellow color than the other formulations. This cured color was not reached with the addition of RGP. However, its inclusion slightly reduced lipid and protein oxidation in salchichón. PC showed high levels of sulfur and terpene levels in a volatile profile, although at a sensory level, only differences in spicy taste were not noticed by panelists. The incorporation of the ingredient could enable the substitution of nitrites with valorized red grape pomace in sausages, although the desirable color achieved with nitrifying salts was not fully attained.

## 1. Introduction

The “salchichón” is a fermented dry-cured sausage which has a prolonged process of drying and ripening before consumption. It is generally made from a mixture of chopped meat (pork and/or beef) and lard, is seasoned with spices such as salt, black pepper, additives (nitrate, nitrite, and antioxidants), and optionally has starter cultures. This process originates physico-chemical [1] and microbial changes, including fermentation, dehydration [2], color changes [3], lipolysis [4], and proteolysis [5]. Oxidation reactions in meat and meat products during processing and storage under conventional settings are common. Maturation favors the formation of the typical aroma compounds of dry-cured meat products, which can be provided by the seasoning added and by the main pathways of lipid-oxidation reactions [6]. However, an excess of lipid oxidation can lead to the formation of hydroperoxides, promoting the generation of some negative volatile compounds, such as some aldehydes, ketones, acids, and alcohols [7]. Additionally, the oxidation process of proteins and amino acids may decrease their bioavailability, digestibility, solubility, and proteolytic activity [5,8]. Oxidation reactions can also adversely change the product’s appearance by oxidizing myoglobin to oxymyoglobin and metmyoglobin and producing brown pigments [9]. Sensory and nutritional attributes, as well as consumer satisfaction, could be negatively altered by these factors.

The addition of nitrate and nitrite salts has a key role in the inhibition of foodborne pathogens, like the Gram-positive spore-forming anaerobic bacteria *Clostridium botulinum* and the most resistant Gram-negative aerobic/facultative anaerobic bacteria (*Escherichia coli* and *Salmonella*), which are inhibited by nitrite salts [10]. On the other hand, nitrifying salts contribute to the development of the typical flavor and red color, also retarding lipid oxidation in cured meat products [11,12,13]. Despite the significant technological and safety goals of nitrites, they can react with secondary amines in the gastrointestinal tract to form carcinogenic N-nitrosamines, which are linked to the incidence of some types of cancer [14].

Consequently, given the potential health risks related to the residual nitrite in meat products, consumers are demanding nitrite-free products that maintain sensory and microbiological quality [13] or technological strategies that involve the applications of natural antioxidants directly to meat and meat products [15]. Researchers and meat industries search for clean labels on the possibility of reducing [16] and substituting [17,18] sodium nitrite. A substantial source of bioactive and natural additives, or their extracts, can be obtained from agro-industrial by-products, thereby creating a circular and sustainable economy.

This interest of consumers in sustainable industrial practice, along with the benefits of reducing industrial waste discharge, increases the economy of reuse systems or valorization strategies. Grape pomace is the main by-product of winemaking, accounting for 62% of organic waste, and mainly consists of skin, pulp, stalk residuals, and seeds. The most common destinations, in Europe, for solid grape residues are land spreading, distillation, incineration, serving as a source of tartaric acid, oil recovery, or animal feeding [19]. Nevertheless, this material contains several compounds (fiber, proteins, fat, and phenolic compounds) with health benefits that may add value to products in the food, cosmetics, or pharmaceutical industries [20]. In addition, this residue may be used for other purposes, such as natural dyes, preservatives, and/or antioxidants in meat products [18,21].

Red grape pomace (RGP) is recognized for its rich content of polyphenols, mainly catechins, epicatechins, gallic acid, and procyanidins, with a multitude of biological properties, such as antioxidant, antimicrobial, or anticancer activities [22,23]. However, its bioactive compounds content is variable, and it is influenced by environmental factors and grape varieties, among others [24]. For better recovery of valuable compounds from grape pomace, many extraction techniques have been studied, such as Soxhlet extraction, maceration, supercritical fluid extraction, subcritical water extraction, and ultrasound-assisted extraction [22,25], but today, these are being replaced by the so-called non-conventional methods. Traditional methods often require lengthy procedures and a variety of specialized solvents. In contrast, new techniques prioritize sustainability and efficient preservation of these valuable components. In this sense, HHP (hydrostatic high-pressure) is considered to be energy-efficient and environmentally friendly in food processing [26,27]. In previous studies, HHP has been described as a suitable technology for the valorization of the red/white wine pomace to produce a possible ingredient where antioxidant and antimicrobial properties—phenolic compounds—could be maintained after processing [28,29]. HHP allowed for a sustainable valorization of grape pomace since no solvents are required, and the whole by-product is reused, thus generating no residues. However, HHP does not always reduce the activity of the polyphenol oxidase, necessitating other hurdles (such as thermal blanching) to stabilize grape pomace [29,30,31].

Several studies have examined the impact of grape pomace as an alternative ingredient to reduce the quantity of nitrite in different meat and meat products. Regarding dry fermented sausages, there are only a few studies that have used grape-seed extract in dry-cured sausage [12], in Spanish “Chorizo” [9,32], in “Cinta Senese” or Italian dry fermented sausage [17,33], and with grape-seed flour in Turkish dry fermented sausage or “Sucuk” [34]. These extracts were found to be effective inhibitors of oxidative reactions, with TBARS (Thiobarbituric acid-reactive substance) values under 1 mg MDA (malondialdehyde) kg^−1^, which is considered as the threshold of sensory perception for lipid oxidation, thus not producing “off-odours” [9]. Moreover, extracts of grape pomace helped to avoid the growth of food pathogens (*Listeria monocytogenes*, *Salmonella* spp., *Staphylococcus aureus*, coliforms, and *E. coli*). Phenolic compounds exhibit antibacterial properties by depriving microbes of essential nutrients like iron and disrupting key microbial processes [15]. They specifically target the microbial cell membrane, causing structural damage and increasing permeability. This allows the phenolic compounds to penetrate the bacterial cell, leading to cell lysis, the release of intracellular ATP, and the loss of vital cellular contents. The antimicrobial and antioxidant properties of phenolic compounds make them viable alternatives to nitrates and nitrites in meat products. A study [17] showed that adding dry grape pomace extract to boiled sausages reduced the nitrite levels while maintaining both microbiological stability and sensory quality. This suggests that grape pomace can be used to lower the amount of curing salts with a toxic potential without affecting the taste or quality of the meat.

Therefore, most of the reported studies deal with the use of polyphenol extracts from grape pomace, seed, or skin to promote antioxidant and antimicrobial activity in meat products. Nevertheless, the integral use of RGP provides the advantage of incorporating dietary fibers together with phenolic compounds. This is particularly beneficial since dietary fibers were identified as predominant compounds in red grape pomace [23]. To the best of our knowledge, no previous studies have investigated the impact of incorporating integral red grape pomace (RGP) stabilized with the HHP technique for preservation in dry-cured sausages. Therefore, the main objective of this study is to evaluate the incorporation of valorized RGP from Tempranillo var. into dry-cured sausages to improve their preservation and replace the use of synthetic additives like nitrifying salts.

## 2. Materials and Methods

### 2.1. Manufacture of the Ingredient from Red Grape Pomace (RGP)

In September 2022, a wine manufacturing company located in Santa Marta de los Barros (Badajoz, Spain) provided red grape pomace (cv *Tempranillo*). About 5 kg of red grape pomace were collected (“initial” pomace). The process includes thermal blanching, grounding, vacuum packaging, and finally, HHP. Before high-pressure processing (600 MPa/5 min), RGP was thermally blanched (TB) to inactivate the PPO enzyme by an exhausting unit (Chaconsa, Murcia, Spain) applying steam at 103 °C for 1 min of residence. These conditions were chosen based on our previous experiments, where scalding was applied for durations ranging from 1 to 5 min, followed by an evaluation of polyphenol oxidase enzyme activity and total phenol content. The enzyme activity of the PPO was decreased completely at 1 min of blanching with a stabilized phenol content [31].

The thermally blanched RGP was vacuum packaged and frozen at −18 °C for 24 h and then crushed using an Ultra Centrifugal Mill (RETSCH ZM200, Haan, Germany) until achieving a fine powder. This powder of RGP was vacuum packaged into Eurobag plastic bags with the following characteristics: polyamide/polyethylene 20/100, oxygen permeability of 50 cm^3^ m^−2^ 24 h^−1^, 0% relative humidity, and 120 µm of thickness. Immediately, the packaged ground pomace was processed in semi-industrial equipment (6000/55, Hiperbaric, S.A., Burgos, Spain), with a container capacity of 55 L at 600 MPa for 5 min at 16 °C of initial temperature of the water (TB + HHP pomace). The ingredient from red grape pomace was stored at −80 °C until being added in different proportions into dry-cured sausages.

To perform the analysis of the initial pomace and the TB pomace samples, they were finely crushed after being well-frozen in a domestic blender Thermomix TM-6 (Vorwerk, Vorwerk, Germany) at maximum speed for 1–2 min. In the same way, it was carried out for the TB + HHP pomace.

### 2.2. Dry-Cured Sausages Preparation

Meat (12 kg) for the manufacture of the dry-cured sausages was acquired in a local market. Minced meat (60% minced pork, 40% minced fresh bacon) was mixed with the following ingredients: 12 g kg^−1^ fresh minced garlic, 20 g kg^−1^ sodium chloride, 1 g kg^−1^ of nutmeg, 1 g kg^−1^ white pepper and 1 g kg^−1^ black pepper. Masses of 3 kg were prepared consecutively for each formulation: negative control (NC), positive control (PC), low-level pomace (LL), and high-level pomace (HL). First, the NC batch (3 kg) was prepared with the previous formulation. The PC batch (3 kg) was prepared with the previous mixture and by adding synthetic additives (0.5 g kg^−1^ of acid L-ascorbic (Laffort, Valladolid, Spain) and 0.1 g kg^−1^ of sodium nitrite (Panreac, Barcelona, Spain)). The LL batch was prepared with the initial recipe and the RGP ingredient (0.5% *w*/*w*), and the HL batch was made with the initial recipe and the RGP ingredient (1% *w*/*w*). Levels of pomace were chosen according to previous studies, taking the highest levels that were not rejected at the sensory level [31]. All the masses were mixed using an automatic vacuum mixer (Talleres Cato, Barcelona, Spain) for 5 min and stored in refrigeration for 1–2 h, and then, these were stuffed into a 45–50 mm diameter natural dried pork casing.

**Figure 1:**
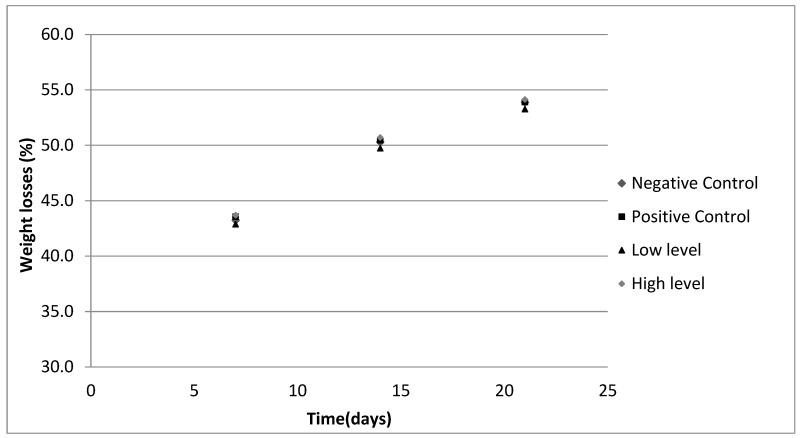
Weight loss (%) of dry-cured sausages during maturation.

Once the sausages were formed, they were dried in the industrial dryer of our pilot plant at temperatures between 12 °C and 14 °C and 85% RH for 21 days. The weight losses at the end of the maturation process were between 43.3–54.1% (Figure 1). Seven sausages per batch were prepared, although 5 per batch were analyzed. Therefore, a total of 20 sausages were evaluated. At the end of the maturation, each sausage reached a weight of around 190 g.

### 2.3. Analysis of the Ingredient from RGP

The phenolic compound content was determined by the Folin–Ciocalteu reagent-based colorimetric assay. The absorbance was measured at 765 nm using a Thermo-Evolution 201 spectrophotometer (Fisher Scientific SL, Madrid, Spain). A calibration curve using Gallic acid as the reference standard was generated. The total phenolic content was expressed as the Gallic acid equivalents per sample (wet base) (mg GAE 100 g^−1^).

Polyphenol oxidase (PPO) activity was measured at 420 nm and 25 °C for 3 min in a Thermo Scientific Evolution UV Vis spectrophotometer (Fisher Scientific SL, 187 Madrid, Spain), in a kinetic model. The results were expressed as a percentage of activity with respect to the control samples.

The composition of the initial pomace was analyzed in 3 independent vacuum-packaged bags. For pH and water activity measurements of the pomace, a pHmeter Crison pH 25 + (Crison, Barcelona, Spain) and a Novasina (Labmaster, Lachen, Switzerland) were used. Moisture and protein analyses were conducted according to the AOAC methodology [35]. The fat content was assessed by the Folch method [36] and fiber according to the modified Southgate method [37], all of them in wet base (WB). Fatty acid methyl esters (FAMEs) from the initial pomace were analyzed using an Agilent 6890 gas chromatograph (Agilent Technologies, Santa Clara, CA, USA), equipped with a flame ionization detector (FID) and a fused silica column (60 mm length, 0.25 mm inner diameter, and 0.25 m film thickness). The injector and detector temperatures were 260 °C and 280 °C, respectively. The column oven temperature was raised to 220 °C on a ramp temperature, and helium was used as a carrier gas, with a constant flow of 1.2 mL min^−1^ and make-up of 25 mL min^−1^. The injection mode was used with a split ratio of 1:100. Individual FAME identification was carried out on the basis of Sigma standards (Supelco 37 component FAME mix standard, Sigma Aldrich, St. Louis, MO, USA) compared with the retention times obtained. The results are expressed as a percentage of total fatty acid methyl esters.

For microbiological analysis, 10 g of RPG was aseptically weighted in a sterile plastic bag and homogenized with 90 mL of a sterile solution peptone water (Merck, Darmstadt, Germany) in a masticator blender (Stomacher 400 Circulator, Seward, West Sussex, UK) at a 1/10 dilution (*w*/*v*). Mesophilic, molds and yeasts and *Enterobacteriaceae* counts of RGP were determined. All microbial counts were expressed as the log of colony-forming units (CFU) per g of sample weight (log CFU g^−1^).

### 2.4. Analysis of Dry-Cured Sausage

The analysis of pH, proximate composition, water activity, and FAMEs followed the procedure previously described.

For the microbiological analysis, 10 g of sausage were taken and homogenized with 90 mL of sterile peptone water. Serial decimal dilutions were prepared in sterile peptone water, and 1 mL of each sample was spread on suitable culture media. Mesophilic aerobic counts were analyzed by a standard Plate Count Agar (Merck, 1.07881), and the plates were incubated at 30 °C for 72 h. Psychrophilic counts were determined on Plate Count Agar (Merck, 1.07881) after incubation at 7 °C for 10 days. Lactic Acid Bacteria (LAB) were incubated on Man Rogosa Sharpe Agar (MRSA, Scharlau, Barcelona, Spain) at 37 °C for 72 h; anaerobic sulfite-reducing *Clostridium* spp. were incubated on Tryptose Sulfite Cycloserine Agar (Merck, 1.10235) at 37 °C for 24 h. Staphylococcus aureus was determined on Baird Parker Agar (Merck, 1.05406) after incubation at 37 °C for 24–48 h. The total coliforms and *E. coli* and were incubated on Chromocult Agar (Merck, 1.10426) at 37 °C for 24–48 h. The molds and yeasts were incubated at 25 °C for 4–5 days. Finally, *Salmonella* and *L. monocytogenes* were determined according to ISO 6579, 1993 and ISO 11290-1, 1996, respectively. Microbial counts were expressed as the log of colony-forming units (CFU) per g of sample weight (log CFU g^−1^) and the absence of *L. monocytogenes* or *Salmonella* spp. in 25 g of sample.

For the instrumental color, the color coordinates lightness (L*), redness (a* red–green axis), and yellowness (b* yellow–blue axis) in the CIE Lab color space were analyzed. In addition, the Hue angle was calculated (h° = tan^−1^ (b*/a*)), as well as the saturation index or Chroma (C*) (C = (a*^2^ + b*^2^)^0.5^). One slice of one-centimeter thickness of the sample was prepared. Two readings were recorded (one on each side of the slice), and a value was determined as the mean of the readings.

For oxidative stability, lipid oxidation was assessed by thiobarbituric acid reactive substances (TBA-RS) according to Sørensen and Jørgensen (1996) [38]. TBARS values were calculated from the standard (1,1,3,3-Tetraethoxypropane, TEP) curve, and the results were expressed as mg of malondialdehyde per kg of sample (mg MDA kg^−1^). Protein oxidation was obtained by measuring the carbonyl groups formed during incubation with 2,4-Dinitrophenylhydrazine (DNPH) in 2 N HCl following the method described by Oliver, Ahn, Moerman, Goldstein, and Stadtman (1987) [39]. Protein oxidation was expressed as nmol carbonyls mg protein^−1^.

For sensory evaluation, a trained sensory panel was formed by eight judges with specific training in the sensory analysis of dry-cured products. Two slices from each dry-cured sausage were presented to each panelist. At each session, one dry-cured sausage from each batch was analyzed, so a total of five sessions were required. The following descriptors were examined: lean color (uncured–cured) and fat color (white–yellow), intensity “salchichón” odor, unpleasant odor, hardness, juiciness, unpleasant texture, salty, spicy, aromatic intensity, and unpleasant taste/flavor. The intensity of each parameter was calculated from 0 = low intensity to 10 points = high intensity. Panelists assessed the different parameters by a quantitative–descriptive analysis with a structured scale (0–10). Data were collected using the FIZZ software, 2.45 A version (sensory analysis and computer test management) (Biosystemes, Couternon, France). All sessions were conducted at room temperature in a sensory room equipped with white, fluorescent lighting. About 100 mL of water at room temperature were provided to the panelists between samples.

For the volatile compounds, two grams of ground sausages were placed into a 20 mL vial (screw-capped with a Teflon-silicone septum). A 1 cm 50/30 μm DVB/CAR/PDMS SPME fiber (Supelco, Bellefonte, PA, USA) was utilized for the analysis of volatile compounds in the headspace of the previously prepared vial. The fiber was exposed to the headspace at 37 °C for 30 min.

For the separation of volatile compounds, a Varian CP-3800 gas chromatograph with a CombiPAL autosampler (CTC Analytics, Zwingen, Switzerland) and with an HP-5 capillary column (30 m × 0.32 mm × 0.25 μm; Agilent Technology, Santa Clara, CA, USA) was used. The injection port was at 270 °C, and the oven temperature was held at 35 °C for 10 min, increased to 250 °C (7 °C min^−1^), and held for 5 min, with a total running time of 45 min. The temperature of the transfer line, trap, and manifold were 280 °C, 200 °C, and 60 °C, respectively. Identification was carried out in a Varian Saturn 2200 MS mass spectrometer (Varian Inc., Palo Alto, CA, USA), and mass spectra were obtained by electronic impact at 70 eV, with one scan s^−1^ over the 40–300 *m*/*z* range.

The volatiles were identified by comparing their mass spectra and linear retention indexes (LRI) with commercial standards (Sigma-Aldrich, St. Louis, MO, USA) or by mass spectra identification using the NIST library (Agilent MSD Chemstation E.02.01.1177 software). The concentration of compounds was estimated by using the internal standard (4-methyl-1-pentanol), which was added to each sample (final concentration: 1.22 mg kg^−1^) and expressed as μg kg^−1^.

### 2.5. Statistical Analysis

The analyses of the RGP were performed in triplicate (three bags per batch), and the mean values and their standard deviations (SD) were calculated. In the assay of dry-cured sausages, five samples per batch were evaluated. In order to evaluate the changes during processing in grape pomace (Table 1), a Student’s *t*-test was applied between initial pomace vs. TB pomace (*p*-value initial-TB); another Student’s *t*-test was applied between TB vs. HHP pomace (*p*-value TB-HHP). Finally, an ANOVA was applied to evaluate differences among the three groups (*p*-value treatments; and then a Tukey’s HSD test was applied when differences were significant). One-way ANOVA was employed to find differences among treatments using the SPSS 21.0 statistical program (SPSS Inc., Chicago, IL, USA). If the ANOVA detected significant differences between mean values, these were compared using Tukey’s test (*p* < 0.05). Principal component analysis (PCA) was carried out with instrumental color and oxidative parameters to evaluate the relationships among the samples of the four groups.

## 3. Results and Discussion

### 3.1. Valorization Process and Composition of the Red Grape Pomace

The valorization process consisted of a thermal blanching, crushing, and a hydrostatic high pressure (HHP) treatment. Previous studies were carried out to know the effect of HHP on RGP and demonstrated that HHP treatment does not reduce PPO activity, resulting in a reduction of bioactive compounds in the valorized products during storage [29,30]. For that reason, the grape pomace was thermally blanched (TB) with the purpose of inactivating the PPO enzyme before HHP treatment. The one-way analysis of the variance (Table 1) showed a complete inactivation of PPO after TB (1 min, 103 °C), and it remained inactivated until the end of the valorization process.

Table 1 shows the total phenolic compound content (PPC) during the valorization process, indicating that TB significantly increased PPC. This initial rise in the PPC of the RGP ingredient aligns with previous research, demonstrating that blanching vegetables increases their phenol content [40]. Some studies have attributed the reported PPC increase to the reduction and inactivation of polyphenol oxidase, along with the release of bound phenolic acids resulting from the breakdown of cellular constituents in the plant cell walls of the leafy vegetable [14]. On the other hand, a significant PPC reduction is observed on the RGP ingredient (TB + HHP) after HHP treatment, which is an unexpected result. Previous studies reported that HHP maintained or even increased the extraction of PCC in grape pomace [41,42]. This increase is induced by HHP through structural changes in the cell matrices, leading to the extraction of phenolic compounds [41]. In the same vein, a parallel study in our research group reported slight non-significant reductions in PCC in “Tempranillo” red grape pomace after treatments at 600 MPa for 5 min [30]. Similarly, the valorization process by HHP of white grape pomace has been recently published [31], and an important increase in PCC was observed after TB. However, the levels were well-preserved after HHP. Compared to those previous studies, in our study, the equipment used to crush the pomace was different, as it allowed the obtaining of a fine powder. The grapes were scalded and then frozen before crushing, resulting in an intense reduction of particle size before HHP treatment. This reduction in particle size was more intense than that obtained with white grape pomace, potentially facilitating the degradation of phenolic compounds by exposition to the air. At this point, an improvement in crushing methodology would be required to avoid the loss of phenolic compounds before HHP treatment.

The proximate composition of the percentage wet base (%WB), pH, and aw of the RGP ingredients are shown in Table 2.

The low pH and aw could provide long-term stability for the valorized pomace, as indicated by previous studies, which suggest a shelf life of at least 9 months [30]. This fact is particularly important for a seasonal by-product like this. Additionally, the low pH (<4.5) in the RGP ingredient also contributes to the great stability of anthocyanins [43]. The fiber was the major component in the RPG ingredient (50.3 ± 1.5%), even above moisture (39.6 ± 3.5%), followed by lipids (5.1 ± 0.9%) and proteins (3.4 ± 0.1%). All described values are within the range of those reported by Antonic et al. (2020) [23]. These data are difficult to compare, since the results of the proximate composition are often given on a dry basis or derived from flour of grape pomace [19]. And sometimes, only skins are used without seeds or stalk remains [24].

The major fatty acids found in our study were linoleic acid (C18:2 ω6, 66.0%), oleic acid (C18:1 ω9, 18.1%), palmitic acid (C16:0, 9.8%), stearic acid (C18:0, 4.1%), and linolenic acid (C18:3, 1.2%) (Table 2). The amounts of these major fatty acids were in the intervals of values indicated for grape-seed oil in previous studies. The lipid fraction, derived from the seeds, is rich in unsaturated fatty acids and powerful antioxidants, such as vitamin E [23].

From the point of view of microbial counts, the application of HHP treatment (600 MPa, 5 min) effectively reduced microbial counts in the RGP ingredient (Table 2). Microbial counts are within acceptable ranges. Previous research about the effect of HHP treatments on red and white grape pomace found that processing conditions of 600 MPa for 5 min reduced the microbial population and allowed a long shelf-life for the processed pomace [29].

### 3.2. Effect of the Incorporation of the Valorized Pomace Ingredient in Dry-Cured Sausages

Table 3 shows the proximate composition and fatty acids profile of dry-cured sausage. In general, dry-cured sausages showed a lower content of fat and moisture and a higher protein content than other similar dry-cured products [44]. Fatty acid composition presented high levels of oleic and polyunsaturated fatty acids, such as linoleic acid (C18:2) and linolenic acid (C18:3).

The percentages shown in Table 3 are higher than those reported in other studies on dry fermented sausages [1,2,4], but our results are like those found by Moretti et al. (2004) [45] in a typical Sicilian salami under ripening-room conditions. Differences in fatty acid profile could be attributed to the different meat cuts utilized [7], the way of manufacture of the “salchichón” [1,2], or the differences in the feeding composition [44]. The fatty acid composition could affect the stability of dry-cured meat products, since unsaturated fatty acids are easily oxidized. Sausages with an RGP ingredient could not be considered fiber-enriched foods because the calculated level of fiber added from RGP is lower than 6 g per 100 g. This is the limit required by European legislation (CE Nº1924/2006). However, in the present study, the objective would not be to increase the fiber levels but to obtain a substitute for nitrites through the incorporation of bioactive compounds from the pomace into the sausage.

The moisture and aw of dry-cured sausages (Table 4) were comparable to that reported in similar products [1,2,3,9,12,17,33,34]. Aw values of <0.89 contribute to controlling pathogenic organism growth [46]. NC sausages presented higher pH values than the rest of the groups, with PC being the batch with the lowest pH value. The pH values were consistent with those usually reported for dry-cured sausages without a natural antioxidant and with nitrite salts after fermentation and after about 3–5 weeks of dry ripening [1,2,3,7,44]. After fermentation, the pH values decrease due to the production of organic acids by lactic acid bacteria, and during the drying and ripening period, the pH increases again due to the release of peptides, amino acids, and ammonium from proteolytic reactions [9,12,17,32,33,34]. Dry-cured salchichón generally has a long shelf-life, which is determined by the low water activity and an acidic pH. The development of LAB during ripening produces lactic acid, which is responsible for the low pH of these products, which provides a microbiologically safe product. The combination of an acid pH, low moisture content, and water activity avoids the growth of other spoilage or pathogen microorganisms, so an adequate development of the fermentation and drying processes are critical points in these traditional products. In our study, the addition of the RGP ingredient in salchichón maintained the pH at similar levels as typical commercial dry-cured meat products, which is important to reach an adequate fermentation.

Microbiological counts of the dry-cured sausages are presented in Table 4. The initial counts (log CFU g−1) in masses before drying were mesophilic counts (7.0 ± 0.1), LAB (4.9 ± 0.1), *Clostridium perfringens* (0.9 ± 0.1), *Staphylococcus aureus* (1.9 ± 0.1), total coliforms (4.7 ± 0.2), *Escherichia coli* (2.3 ± 0.5), and molds and yeast counts (>4). The raw mixtures were in microbiological safety conditions, and these counts are considered in the range reported in comparable studies [2,7,12,32]. In the dry-cured sausages (Table 4), the counts (log CFU g^−1^) of mesophilic and psychrophilic presented similar levels in all formulations of dry-cured sausages. However, the counts of LAB were lower in the NC group compared to the other groups, which agrees with the higher pH observed in these dry-cured sausages after maturation. The final pH of a fermented meat product is a consequence of the development of LAB during the maturation process, which is typical of the microbiota of fermented sausages [45]. Lactic aerobic bacteria produce lactic acid during the maturation process, with decreases in pH and the inhibition of microbial growth of other species and pathogenic bacteria, especially *Staphylococcus aureus* [7] or *Enterobacteriaceae* [2]. Therefore, high growth of LAB is linked with a low final pH in fermented meat products, and this low pH helps stabilize the fermented meat product microbiologically by inhibiting the growth of other pathogens [12].

The counts of *C. perfringens*, *S. aureus*, total coliforms, *E. coli,* and molds and yeasts were similar in all groups (*p* < 0.05), and they were not affected by the formulation. The counts of dry-cured sausages were in the normal range for fermented meat products, and they were in line with those reported in Sicilian Salami (Moretti et al., 2004), traditional dry-cured sausages [7,45], the dry-cured sausage “salchichón”, or “chorizo” with grape pomace or its extracts [9,12,32]. Finally, *Salmonella* and *Listeria* were not detected in any group (absence in 25 g), as *S. aureus*, *E. coli*, total coliforms, and C. perfringens were also absent or inferior to the limit established by regulation (CE) nº2073/2005.

According to a previous in vitro study, the antimicrobial activity of extracts from grape seeds (*Vitis vinifera* L.) was estimated against *E. coli* and *Listeria innocua* [47], and this activity could remain unchanged after HHP [30]. This technology was utilized for the stabilization of the RGP. In fresh burgers, the ingredient from white pomace presented some antimicrobial effect, since its addition to the burger reduced the counts of molds and yeasts and total coliform [31]. In the case of dry-cured sausages, the antimicrobial effect was not evident, since most counts were similar in all groups. However, the inclusion of RGP favored the acidification of the product and the levels of LAB were comparable to the found in the PC, which was manufactured with nitrifying salts and ascorbic acid.

Nitrites control the proliferation of bacteria that causes meat spoilage [48], and they also contribute to the control of other pathogenic microorganisms, such as *E. coli* O157:H7, *S. aureus*, and *Salmonella* [49]. The protective effect against pathogens by the addition of ascorbic acid and nitrites was not perceived in PC sausages. It should be considered that other types of studies, such as challenge tests, would be required to further investigate this effect. At the industrial level, ascorbic acid is generally added together with nitrifying salts to increase the stability of nitrites. Additionally, this acid influences reducing pH, as well as exhibiting antioxidant and antimicrobial activities [10].

Significant changes in color were detected among dry-cured sausages, and only the lightness was similar in all groups (Table 5). The PC group presented significantly higher a* and lower b* than the NC group. In general, the addition of the RGP ingredient to dry-cured sausages had no significant effect on color variation compared to the NC. However, a significantly lower chroma was found in the HL compared to the NC group. In addition, the redness values of dry-cured sausage with RGP (LL, HL) and NC were also similar, since the levels of pomace added were low enough to avoid color modifications in the meat product. In this line, previous studies have also reported that the L* values of dry-cured sausage were not affected by the incorporation of grape-seed extracts [17,32,33]. The redness value was like that reported by Kurt (2016) [34] in Turkish dry-cured sausage with grape-seed flour. This similarity might be justified by the color pigments present in grapes.

The effect of the nitrite-enhancing a* value in PC was somewhat expected, considering the role that nitrites play in the formation of the red curing pigment nitrosomyoglobin [13]. Moreover, the PC batch exhibited a significant difference compared to the NC in terms of the b* value, which was the highest. These variations in instrumental color coordinates also resulted in changes in hue and chrome values. In line with our results, Higuero et al. (2020) [11] reported increasing values of CIE a* and reduced values of CIE b* in dry-cured loin due to the addition of nitrifying salts. Therefore, nitrifying salts are not only responsible for the higher redness but also for the lower yellowness of the dry-cured meat products.

The mean values of TBA-RS were low (<0.5 mg MDA kg^−1^) for all groups, indicating low lipid oxidation during the drying process (Table 5). The PC batch showed a significantly lower TBA value compared to the NC batch. Both levels of the RGP ingredient (LL and HL batches) presented intermediate values, which could indicate a slight tendency to decrease lipid oxidation. The mechanism by which nitrite inhibits lipid oxidation is clearly defined, including the formation of nitrosyl–myoglobin (MbNO) and nitric oxide ferrous complexes, the synthesis of S-nitrosocysteine, and the inhibition of the Fenton reaction due to the neutralization of the release of Fe^2+^ from myoglobin [13]. Previous studies have reported an intense antioxidant effect from the addition of grape-seed extracts [9,17] and grape-seed flour [34]. A possible explanation for these differences regarding the antioxidant effect of RGP could be that the mentioned studies have added extracts from the grape seed, in which the concentration of polyphenols would be higher than in our valorized by-products (skin, seeds, and stalks). However, in a previous study, the addition of white grape pomace to burgers at different levels of inclusion (0.5, 1, or 3%) significantly decreased lipid oxidation compared to a negative control group without pomace, even compared to a positive control group including sulfites [31].

The addition of the RGP ingredient had a significant impact on protein oxidation, as measured by the carbonyl content (Table 5). Samples from the LL batch (0.5%) and HL batch (1%) presented significantly lower carbonyl contents than the samples from the NC group. However, the addition of nitrites (PC) did not lead to a decrease in protein oxidation in the dry-cured sausages. The effect of nitrite on protein oxidation is unclear, and there are contradictory results regarding its impact on protein carbonylation [5]. Depending on the concentration, sodium nitrite has both anti- and pro-oxidant outcomes on protein oxidation in meat products [50]. Regarding the observed effect of the RGP ingredient in our samples, this is consistent with the study by Yu et al. (2013) [51] who incorporated grape-seed phenolic compounds into Chinese-style sausage. In this study, the inhibition of protein oxidation was attributed to the prevention of metmyoglobin formation by the competitive chelation of iron from myoglobin. However, there are also studies that do not describe this effect and even report an increase in carbonyl groups compared to sausages including nitrites or phenolic compounds [9,12,17,32,34], despite the influence of phenolic compounds on protein oxidation due to the chemical structure of the phenolic compounds and their interactions with the myofibrillar proteins [5]. In our study, the effect of the addition of the RGP ingredient on decreasing protein oxidation was somewhat unexpected, since it presented more effectivity than nitrifying salts. A previous study on model systems reported that protein oxidation is a complex group of reactions, and nitrite can act as both an antioxidant and a pro-oxidant on proteins [8]. In line with our results, sliced dry-cured loin manufactured with nitrites presented lower values of carbonyls with respect to that free of nitrites [11].

**Figure 2:**
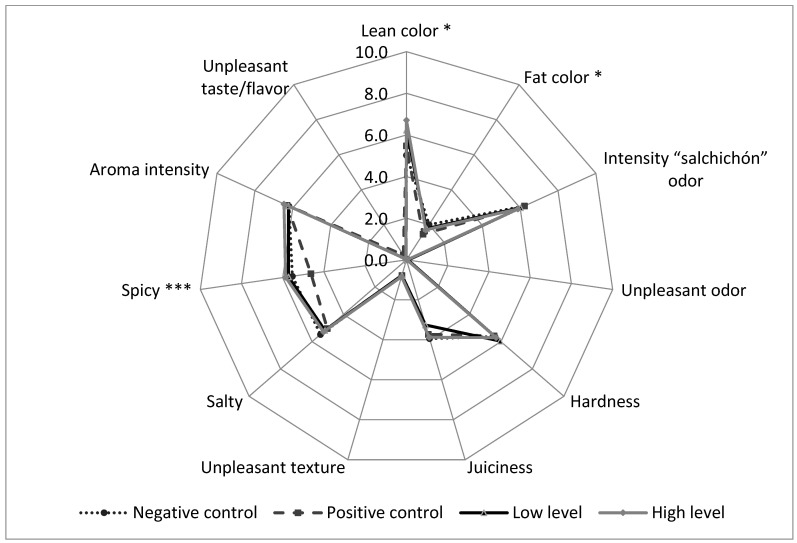
Radar chart for sensorial profile of dry-cured sausages. Negative control (sausages manufactured without nitrites); positive control (sausages manufactured with nitrites); low level (sausages manufactured with 0.5% (*w*/*w*) red wine pomace); high level (sausages manufactured with 1% (*w*/*w*) red wine pomace). ns. (non-significant differences). * *p* < 0.05; *** *p* < 0.001.

Figure 2 displays the results of the sensory analysis of dry-cured sausages from different batches. There were no statistically significant differences in the attributes assessed by the panel for the addition of the RPG ingredient, except for the lean and fat color. Appearance descriptors showed higher scores regarding the intensity of lean color in the PC group compared to the NC group, while RGP (LL and HL) showed the highest lean color intensity. In addition, PC presented significant differences in fat color, since the yellowness of fat was lower than in the other groups. These results are consistent with the instrumental color differences (Table 5). On the other hand, the spicy taste was lower in PC than in other groups, although no explanation has been found for these results. Nitrites act as antioxidants and modulate the aroma and taste formation in dry-cured products [14]. Some panelists noticed an excess of spicy taste in the product, which could be modulated by the inclusion of nitrites. Therefore, the use of nitrites produced a positive effect at the sensory level which was not achieved by the addition of pomace.

It is important to highlight that the use of the RPG ingredient did not negatively affect the texture and aroma characteristics in the sensory analysis of the sausages. The tasters did not find any unpleasant texture, odor, or flavor/taste in any of the groups, regardless of the recipe used. This is particularly important in the case of the groups prepared with RPG ingredients (LL and HL), which provide a significant content of polyphenols (Table 1). A parallel study with white grape pomace [31], produced by the same methodology, which was added into burgers, found that high levels of the ingredient reduced the acceptability of burgers due to problems with the texture, since the pomace was perceived during chewing. In the current study, pomace was more intensely ground, and the mentioned texture problems have been solved (*p*-value for texture >0.05). But, this has led to a significant reduction of phenolic compounds in the ingredient (Table 1). Furthermore, this fact may have reduced the bioactivity of the ingredient, since as mentioned, the antioxidant effect on salchichón was not intense.

Dry-cured sausages from different groups presented significant differences in 15 of the 49 volatile compounds isolated (Table 6). Volatile compounds were grouped into sulfur compounds (9 compounds), terpenes (19 compounds), alcohols (8 compounds), aldehydes (4 compounds), ketones (3 compounds), lineal hydrocarbons (3 compounds), and aromatic hydrocarbons (3 compounds).

Terpenes and sulfur compounds were the most abundant groups of the volatile compounds isolated. Terpenes represented between 43–51%, while sulfur compounds represented between 32–36% with respect to the total compounds. The highest percentages were found in the PC group (51 and 36%), while the NC and the groups with pomace presented similar levels. On the other hand, the rest of the chemical groups represented minor compounds, such as alcohols (4–10%), ketones (4–5%), aldehydes (2–3%), lineal hydrocarbons (2–11%), or aromatic hydrocarbons (1%).

Despite the difficulties in establishing the origin of some volatile compounds, they could be grouped according to their most probable origin. In dry-cured sausages, most volatile compounds, like terpenes and sulfur, could be derived from the seasoning (in this case, garlic, pepper, and nutmeg) added during the manufacturing process. The second group would be the lipid-derived compounds, like some aldehydes, ketones, and alcohols. In addition, some volatile compounds would be originated from the Maillard reaction, such as Strecker aldehydes and hydroxyketones. In fermented dry-cured meat products, microorganisms also participate in the fermentation and in the degradation of amino acids, like some sulfur compounds. Amino acid degradation can also lead to aromatic aldehydes, such as benzaldehyde or benzene acetaldehyde [6].

The three most abundant compounds isolated in the headspace of dry-cured sausages were diallyl disulfide (a sulfur compound), followed by two terpenes, namely α- terpinene and limonene. These three compounds did not present significant differences among formulations. According to their origin, the lack of significant differences agrees with the similar amounts of garlic, nutmeg, white pepper, and black pepper added to each formulation.

In general, the incorporation of the RGP ingredient did not affect the aroma of the dry-cured meat products, which agrees with the sensory analysis. The most abundant compounds isolated in the valorized pomace ingredient (Table 7) were 2,2,4,6,6-pentamethylheptane, ethyl decanoate, ethyl octanoate, phenylethyl alcohol, acetic acid, and 3-methyl, 1-butanol. Among these compounds, only 2,2,4,6,6-pentamethylheptane, phenylethyl alcohol, and 3-methyl, 1-butanol were also isolated in dry-cured sausages (Table 6), and they were not isolated in high quantities in the batches manufactured with the RGP ingredient.

Sulfur compounds produce a significant impact on the global meat aroma in dry-cured meat products. These compounds produce vegetable and garlic notes, but some compounds, like dimethyl disulfide, could be also derived from amino acids degradation. And it has been associated with meaty odor notes [6]. Since garlic is a potent aromatic ingredient, sulfur compounds derived from allicin, characteristic of garlic aroma [52], likely contribute significantly to the overall aroma of dry-cured sausages. Three sulfur compounds (methyl 2-propenyl disulfide (methyl allyl disulfide), allyl methyl sulfide, and dimethyldisulfide) were more abundant in PC sausages with respect to the other groups. Dimethyl disulfide is a compound that originated from the degradation of methionine and adds savory flavors [6]. Dimethyl disulfide is considered a key odorant in dry-cured ham [53], but it has been also isolated in garlic [52]. It is difficult to explain why these sulfur compounds are in the highest quantities in the PC group. In contrast to our results, other authors have reported that the use of nitrite decreased the abundance of diallyl disulfide, in heat-treated Sucuk, a type of semi-dry fermented sausage [54]. Probably, the differences in the manufacturing processes between both products would explain these dissimilarities.

The increase in sulfur compounds in PC sausages could be associated with different causes. Sulfur compounds (in the case of sulfur compounds originated from garlic) may be better preserved by the addition of ascorbic acid and nitrifying salts, since they have antioxidant properties. In addition, the incorporation of additives also affects microbial counts and amino acid degradation, which could, in turn, affect sulfur compounds originating from this pathway. In this line, other authors have also found changes in sulfur compounds (despite their origin from spices) in dry-cured meat products because of changes in the chloride salts addition. In this context, methyl 2-propenyl disulfide and diallyl disulfide were significantly affected by the replacement of chloride salts in pastırma (a traditional Turkish dry-cured meat product) [55]. So, despite these sulfur compounds originating from garlic, their stability could be probably affected by the formulation of the dry-cured products.

A wide variety of terpenes were isolated in dry-cured sausages. Terpenes have well-defined odors in the literature, so α-pinene has been described to add a pine odor. Meanwhile, limonene and carene terpene add lemon notes. α- and β-pinene are two isomers found in nature in essential oils. In 6 of 19 compounds, significant differences were presented among groups. The highest levels of terpenes were in the PC group, specifically L-β-pinene, limonene, terpinolene, and β-bisabolene, all of which originated from spices added during the manufacturing process. Similarly, NC also presented the highest values of limonene, 4-terpineol, α-terpineol, and trans-α-Bergamotene. Aquilani et al. (2018) [17] reported differences in the terpenes content abundances in Cinta Senese dry-fermented sausages with grape seed and chestnut, in combination with 3-hydroxytyrosol, as a substitute for sodium nitrite. However, these changes did not affect the overall aroma profile, since changes in individual compounds are not always globally perceived. This would be in line with our results, since the panelist did not find differences in the sensory analysis regarding odor and taste intensity in the dry-cured meat products (Figure 2). Only the PC group was noticed to be less spicy than the other sausages, which could be correlated with the highest levels of sulfur and terpenes in this group. These compounds may modulate the perception of spicy taste in the sausages.

Three alcohols were most abundantly isolated in NC samples, including ethanol, 2,3-butanediol, and D-mannose, which are compounds related to the effect of microbial fermentation. During the fermentation process, LAB produces lactic acid and small amounts of other compounds, such as ethanol and 2,3-butanediol. The highest levels of these compounds in NC, which presented the lowest LAB counts and the highest pH, could probably be attributed to less developed fermentation in this batch compared to the other groups. These compounds are the initial products of fermentation, which should be transformed into other flavor compounds. In fact, significant correlations were found between these parameters. The LAB counts were negatively correlated with pH (r = −0.611, *p* < 0.01), ethanol (r = −0.640, *p* < 0.01), and 2,3- butanediol (r = −0.571, *p* < 0.01). The proteolytic and lipolytic activities of both microorganisms are essential to the sensory quality of fermented sausages. The highest levels of sugar-like d-mannose in NC sausages would also indicate less fermentation development in this group. The flavor and aroma of fermented meats are a combination of several elements. Lactic acid bacteria produce lactic acid and other compounds. However, to ensure the sensory quality of fermented sausages, the contribution of the proteolytic and lipolytic activities of *Staphylococcus* is fundamental. In a recent paper with an ingredient from white grape pomace (obtained by the same procedure) and added to dry-cured sausages at different levels (0.5% and 3%), the compound 2,3-butanediol was the highest in the 3% pomace sausages. The high level of this compound was related to less juiciness and a defective texture in the sensory analysis of the dry-cured sausage [53]. In the current study, these changes were not noticed when the sensory analysis was performed.

On the other hand, the levels of 1-octen-3-ol and phenylethyl alcohol (both lipid-oxidation-derived compounds) were the highest in NC sausages, which agrees with the highest TBA-RS values observed in this group. In this line, significant positive correlations were found between the TBA-RS values and 1-octen-3-ol (r = +0.453, *p* < 0.05) and phenylethyl alcohol (r = +0.683, *p* < 0.001), which would support the origin of these compounds.

Aldehyde compounds probably originated from amino acids degradation (benzaldehyde and benzeneacetaldehyde) or the Maillard reaction (3-methyl, butanal) and lipid oxidation (nonanal). None of these compounds originated significant differences among formulations.

The use of the RPG ingredient did not negatively affect the volatile profile of the sausages. However, the incorporation of ascorbic acid and nitrifying salts in the PC group favored the highest contents of some terpenes or sulfur compounds. This fact was not reflected in the sensory analysis of sausages, where only significant differences in the spicy taste were reported. The effect of nitrites in flavor formation is not known in depth, but in the case of dry-cured meat products like ham, a clear effect of nitrites on the modulation of lipid-derived compound reactions has been described [48]. However, in fermented products like salchichón, which have a different volatile profile with more importance on spices and microbial fermentation, this effect is not well understood.

## 4. Conclusions

The valorization process for red grape pomace allowed the full utilization of the entire by-product, resulting in an ingredient rich in fiber and phenolic compounds. The ingredient from red grape pomace favored an adequate fermentation of the dry-cured salchichón, since it enhanced the development of lactic acid bacteria and the acidification of the product. Red grape pomace also provided an antioxidant effect, so lipid-oxidation development was lower in the dry-cured product. This fact has a special relevance, since the sale of the sliced product has increased as a consequence of new market trends. Due to the high surface exposure of the product, the development of oxidation reactions is crucial to reach a long shelf-life. In addition, the ingredient did not negatively affect the sensory perception of the sausages. However, the addition of red grape pomace did not provide the bright-red cured color of salchichón caused by nitrifying salts. Future studies should evaluate the possibility of a partial supply of nitrifying salts instead of a total reduction of these additives, to avoid reducing the safety and modifying the appearance of dry-cured products. Research on the pomace valorization process should prioritize preserving phenolic compounds in the ingredient, as this would enhance its effectiveness in preserving dry-cured meat products.

## Figures and Tables

**Table 1:** Changes in the total phenolic compounds content (PPC, mg 100 g^−1^) and polyphenoloxidase (PPO, % percentage respect to the initial) enzyme during the process of manufacture of the ingredient of red grape pomace.

	Initial (1)			TB (2)			TB + HHP (3)			*p*-Value (1–2)	*p*-Value (2–3)	*p*-Value (1–2–3)
PPC (mg 100 g^−1^)	467.6	±	3.3	882.0	±	41.5	379.5	±	43.7	<0.001	<0.001	<0.001
PPO (%)	100.0	±	4.9	0.0	±	0.0	0.0	±	0.0	<0.001	0.341	<0.001

TB: thermal blanching; HHP hydrostatic high pressure; *p*-value (1–2) (Student’s *t*-test between initial purée and TB purée); *p*-value (2–3) (Student’s *t*-test between TB purée and HHP purée); *p*-value (1–2–3) (Tukey’s HSD test between initial, TB, and HHP). Different letters in the same row indicate significant differences in the Tukey test (*p* < 0.05).

**Table 2:** Physical–chemical composition and microbial counts of the valorized red grape pomace (RGP).

Valorized Red Grape Pomace			
pH	4.2	±	0.1
Aw	0.963	±	0.001
Proximate composition (g 100 g^−1^)			
Moisture	39.6	±	3.5
Protein	3.4	±	0.1
Fat	5.1	±	0.9
Fiber	50.3	±	1.5
Fatty acids profile (%)			
C12:0	0.0	±	0.1
C14:0	0.0	±	0.1
C16:0	9.8	±	0.2
C16:1	0.5	±	0.1
C17:1	0.1	±	0.0
C17:1	0.1	±	0.0
C18:0	4.1	±	0.1
C18:1	18.1	±	0.1
C18:2	66.0	±	0.5
C18:3	1.2	±	0.1
C20:0	0.0	±	0.0
C20:1	0.0	±	0.1
Microbial counts (log CFU g^−1^)			
Mesophilic	1.5	±	1.0
Molds and Yeasts	<1	±	0.0
Enterobacteriaceae	<1	±	0.0

**Table 3:** Proximate composition (g 100 g^−1^) and fatty acids profile (%) of dry-cured sausage (“salchichón”) (negative control batch).

	Mean		SD
Moisture	28.1	±	1.5
Protein	40.5	±	1.9
Fat	13.5	±	2.1
C12:0	0.1	±	0.0
C14:0	1.4	±	0.0
C16:0	23.2	±	0.0
C16:1	3.1	±	0.0
C17:0	0.4	±	0.0
C17:1	0.4	±	0.0
C18:0	10.8	±	0.0
C18:1	45.6	±	0.1
C18:2	13.6	±	0.1
C18:3	0.6	±	0.0
C20:0	0.0	±	0.0
C20:1	0.9	±	0.0

**Table 4:** Physicochemical characteristics and microbiological counts of dry-cured sausages “salchichón”.

	Negative Control			Positive Control			Low Level			High Level			*p*-Value
Moisture	28.1	±	1.5	28.3	±	1.1	26.9	±	1.1	27.7	±	1.0	0.302
Aw	0.829	±	0.011	0.833	±	0.013	0.824	±	0.009	0.832	±	0.010	0.599
pH	5.8 a	±	0.1	5.5 c	±	0.0	5.7 b	±	0.0	5.7 b	±	0.0	0.000
Microbial counts (log CFU g^−1^)													
Mesophilic	8.1	±	0.2	7.0	±	3.9	8.4	±	0.1	8.7	±	0.2	0.556
Psychrophilic	8.1	±	0.1	8.2	±	0.2	8.0	±	0.2	8.1	±	0.2	0.323
Lactic acid bacteria	7.9 b	±	0.1	8.7 a	±	0.2	8.6 a	±	0.4	8.6 a	±	0.2	0.000
*Cl. perfringens*	<1			0.9	±	0.1	<1			<1			0.261
*S. aureus*	<2			2.0	±	0.2	<2			1.9	±	0.1	0.391
Total coliforms	<1			<1			<1			<1			-
*E. coli*	<1			<1			<1			<1			-
Molds and yeasts	4.7	±	1.1	5.2	±	0.7	4.5	±	0.2	3.9	±	0.4	0.069

Negative control (NC: sausages manufactured without nitrites/RGP); positive control (PC: sausages manufactured with nitrites); low level (LL: sausages manufactured with 0.5% (*w*/*w*) RGP); high level (sausages manufactured with 1% (*w*/*w*) RGP). Different letters in the same row indicate significant differences in the Tukey test (*p* < 0.05).

**Table 5:** Instrumental color parameters and oxidative parameters (TBA-RS and carbonyls) of dry-cured sausages “salchichón”.

	Negative Control			Positive Control			Low Level			High Level			*p*-Value
Instrumental color													
L*	45.0	±	2.6	44.5	±	1.7	44.4	±	1.1	42.4	±	2.1	0.197
a*	5.0 b	±	1.1	7.7 a	±	1.5	4.4 b	±	0.6	4.0 b	±	0.6	0.000
b*	12.1 a	±	1.7	9.0 b	±	1.7	10.3 ab	±	1.0	9.8 ab	±	1.1	0.019
Chroma	13.2 a	±	1.4	12.0 ab	±	1.0	11.2 ab	±	1.1	10.6 b	±	1.2	0.017
Hue	67.0 a	±	6.2	49.0 b	±	9.8	67.0 a	±	2.3	68.0 a	±	1.7	0.000
Oxidative parameters													
TBA-RS	0.5 a	±	0.2	0.2 b	±	0.0	0.3 ab	±	0.0	0.4 ab	±	0.1	0.013
Carbonyls	3.1 a	±	1.0	2.3 ab	±	0.4	1.8 b	±	0.3	2.1 b	±	0.3	0.016

Negative control (NC: sausages manufactured without nitrites/RGP); positive control (PC: sausages manufactured with nitrites); low level (LL: sausages manufactured with 0.5% (*w*/*w*) RGP); high level (sausages manufactured with 1% (*w*/*w*) RGP). Oxidative parameters: lipid oxidation or TBA-RS (mg MDA kg^−1^), protein oxidation (nmols carbonyls mg protein^−1^). Different letters in the same row indicate significant differences in the Tukey test (*p* < 0.05).

**Table 6:** Effect of different formulations on the headspace volatile compounds (μg kg^−1^) of dry-cured sausage (“salchichón”).

	LRI	Negative Control			Positive Control			Low Level			High Level			*p*-Value	Descriptors ^a^
Sulfur compounds															
Allyl methyl sulfide	678.61	573.5 ab	±	370.1	879.4 a	±	376.4	212.9 b	±	113.7	527.5 ab	±	310.3	0.030	Garlic
Dimethyldisulfide	720.40	123.9 ab	±	71.6	163.4 a	±	71.2	29.1 c	±	16.7	61.6 bc	±	32.1	0.005	Garlic
Allylsulfide	856.13	1021.5	±	782.6	1318.9	±	556.1	470.8	±	167.8	1144.0	±	645.2	0.160	Cabbage
Methyl 2-propenyl disulfide (methyl allyl disulfide)	911.37	2595.8 ab	±	1748.9	4226.2 a	±	2006.8	1130.8 b	±	441.1	2598.8 ab	±	1706.5	0.049	
Diallyl disulfide	1084.33	11,691.2	±	5637.8	10,850.7	±	4436.5	3971.4	±	1505.3	8689.8	±	5081.0	0.061	Garlic
Allyl disulfide	1105.74	865.5	±	532.1	1369.1	±	978.8	356.0	±	151.7	693.6	±	494.1	0.110	
1,3,5-Trithiane	1160.24	12.1	±	10.0	17.6	±	5.1	7.2	±	3.5	12.1	±	7.1	0.165	
Allyl trisulfide	1315.72	98.1	±	51.8	98.9	±	38.9	28.5	±	13.7	65.2	±	46.4	0.043	Pungent sulfur, garlic
Diallyl tetrasulfide	1566.49	63.0	±	34.7	57.2	±	17.7	19.4	±	9.3	43.8	±	28.6	0.057	
Terpenes															
1R-α-Pinene *	933.06	804.4	±	1665.1	328.6	±	135.1	825.8	±	294.0	495.0	±	705.3	0.790	Pine, turpentine
L-β-Pinene *	977.07	3424.8 ab	±	1763.5	4360.0 a	±	2100.3	1230.1 b	±	415.2	2759.0 ab	±	1845.4	0.049	pine, resin, turpentine
β-Myrcene	994.66	177.5	±	387.7	0.0	±	0.0	303.3	±	81.1	673.3	±	495.7	0.208	balsamic, must, spice
α-Phellandrene	1004.95	1157.7	±	747.6	1425.6	±	897.4	337.8	±	117.6	709.8	±	403.7	0.061	dill
α- Terpinene *	1019.19	9485.1	±	4989.5	10,656.7	±	5530.4	3215.3	±	822.7	6977.9	±	458.6	0.075	lemon
Limonene *	1033.14	6443.7	±	3893.3	6682.2	±	3533.9	1994.7	±	548.6	4374.3	±	3067.5	0.092	lemon, orange
γ- Terpinene	1064.63	626.8 a	±	269.9	685.1 a	±	226.3	192.2 b	±	111.2	492.4 ab	±	279.1	0.018	
Terpinolene	1093.76	226.9	±	130.1	226.1	±	210.9	113.1	±	46.2	160.8	±	83.2	0.479	Pine, wood, mint
β-Terpineol	1186.98	1033.6 a	±	529.4	968.3 a	±	272.2	315.9 b	±	93.9	713.0 ab	±	366.0	0.022	must
α-Terpineol	1200.39	94.5 a	±	53.1	83.4 ab	±	22.3	31.0 b	±	9.6	63.4 ab	±	32.0	0.040	oil, anise, mint
δ-Elemene	1355.56	176.0	±	123.3	155.2	±	69.0	45.3	±	14.8	104.1	±	63.1	0.072	wood
α-Copaene	1394.98	240.1	±	163.0	206.4	±	80.7	54.8	±	35.4	150.9	±	88.9	0.056	wood, spice
L-Caryophyllene	1429.80	46.3	±	36.4	38.0	±	16.2	11.9	±	4.7	27.8	±	17.5	0.117	wood, spice
β-Caryophyllene	1443.58	87.3	±	58.9	72.2	±	24.7	22.3	±	8.8	54.4	±	33.2	0.064	wood, spice
trans-α-Bergamotene	1456.64	12.6 a	±	8.0	10.1 ab	±	3.7	3.3 b	±	1.2	8.1 ab	±	4.4	0.052	wood, warm, tea
α-Caryophyllene	1478.68	1112.6	±	834.1	955.9	±	418.4	300.1	±	107.5	688.7	±	421.4	0.107	earth
β-Bisabolene	1530.86	7.3 b	±	2.7	12.7 a	±	3.6	0 c			5.0 b	±	3.6	0.000	balsamic
δ-Cadinene	1548.32	23.3	±	22.0	19.4	±	13.4	3.6	±	5.0	17.6	±	10.4	0.178	thyme, medicine, wood
Caryophyllene oxide	1613.59	15.2	±	11.9	13.1	±	4.3	5.0	±	1.8	12.5	±	7.3	0.189	herb, sweet, spice
Alcohols															
Ethanol	-	897.7 a	±	494.2	386.7 ab	±	290.3	47.7 b	±	65.7	398.2 ab	±	218.2	0.005	sweet
3-Methyl-1-butanol *	714.29	84.3	±	55.0	114.6	±	37.7	56.1	±	11.1	113.3	±	75.5	0.253	whiskey, malt, burnt
2,3-Butanediol *	767.92	3641.1 a	±	1791.2	988.6 b	±	702.6	343.8 b	±	417.5	2649.0 ab	±	2121.9	0.009	fruit, onion
3,4-Dimethyl-2-hexanol	789.78	98.8	±	148.0	367.4	±	127.0	112.9	±	103.8	433.0	±	497.8	0.155	
1-Octen-3-ol	985.90	59.3 a	±	31.1	27.5 ab	±	8.9	13.8 b	±	2.6	31.6 ab	±	27.7	0.027	Herb, earth, molds
Benzyl alcohol	1040.49	299.5	±	108.0	276.4	±	78.3	118.8	±	53.9	239.0	±	145.3	0.057	sweet, flower
Phenylethyl alcohol *	1120.61	324.4 a	±	203.5	136.2 ab	±	64.1	81.8 b	±	26.3	253.1 ab	±	110.4	0.024	honey, spice, rose, lilac
d-Mannose	1206.52	80.6 a	±	47.6	67.5 ab	±	12.7	20.9 b	±	7.0	41.6 ab	±	25.9	0.018	
Aldehydes															
3-Methylbutanal *	639.07	229.8	±	162.3	39.3	±	38.9	62.5	±	38.7	149.4	±	150.0	0.066	cocoa, almond, malt
Benzaldehyde *	962.63	184.4	±	151.1	84.0	±	54.5	86.2	±	43.3	170.3	±	228.5	0.555	almond, burnt sugar
Benzeneacetaldehyde	1048.85	914.7	±	698.7	530.6	±	118.9	311.8	±	152.7	748.4	±	363.0	0.142	Benzeneacetaldehyde
Nonanal *	1111.01	77.2	±	33.5	261.1	±	328.6	40.9	±	18.0	72.8	±	39.5	0.187	fat, citrus, green
Ketones															
3-Hydroxybutanone	686.92	848.9	±	568.8	662.1	±	210.3	258.3	±	145.8	659.1	±	407.9	0.124	
3-Octanone *	1028.26	1570.6	±	1132.3	1382.1	±	686.3	345.4	±	205.5	890.2	±	604.5	0.074	herb, butter, resin
1-Phenylethan-1-one	1073.10	248.1	±	121.6	243.3	±	75.8	100.8	±	41.1	167.4	±	89.9	0.050	
Lineal hydrocarbons															
2-Methylheptane	743.57	31.1	±	43.0	36.3	±	21.9	107.6	±	63.7	161.1	±	185.6	0.172	
3-Ethylhexane	752.17	33.4	±	60.9	34.4	±	31.0	136.8	±	90.1	205.3	±	242.4	0.159	
2,2,4,6,6-Pentamethylheptane	991.59	1117.6	±	954.4	1085.8	±	523.2	1896.0	±	1141.7	2561.3	±	2259.1	0.306	
Aromatic hydrocarbons															
O-Methyleugenol	1421.26	332.3 a	±	205.7	289.2 ab	±	82.2	86.5 b	±	50.3	226.3 ab	±	119.2	0.040	clove, honey
Myristicin	1544.97	250.8	±	154.9	214.6	±	62.0	77.1	±	24.2	170.0	±	93.6	0.060	spice, warm, balsamic
Elemicin	1576.72	54.8 a	±	34.9	46.9 ab	±	13.1	16.0 b	±	5.0	36.9 ab	±	19.8	0.055	spice, flower

* The identification of the compound was carried out by the mass spectrum and LRI identical with a commercial standard compound. Different letters in the same row indicate significant differences in the Tukey test (*p* < 0.05). ^a^ Flavor descriptors from the Cornell University Flavornet (http://www.flavornet.org/flavornet.html (accesed on 28 September 2024)) and the Good Scents Company (http://www.thegoodscentscompany.com/index.html (accesed on 28 September 2024)).

**Table 7:** Volatile compounds (μg kg^−1^) isolated in the headspace of the valorized ingredient from red grape pomace.

		LRI	Ingredient Red Grape Pomace			Descriptors ^a^
Alcohols	Ethanol *	-	1014.4	±	416.6	sweet
	3-Methyl-1-butanol *	715.16	2173.3	±	1185.8	whiskey, malt, burnt
	2,3-Butanediol *	810.65	462.5	±	409.3	fruit, onion
	1-Hexanol *	873.64	524.3	±	30.5	resin, flower, green
	Benzyl Alcohol *	1034.79	328.3	±	64.6	sweet, flower
	Phenylethyl alcohol *	1113.88	3043.5	±	100.3	honey, spice, rose, lilac
Aldehydes	Hexanal	790.34	316.0	±	7.5	grass, tallow, fat
	Benzaldehyde *	958.98	70.7	±	26.2	almond, burnt sugar
	Phenyl acetaldehyde	1043.62	48.7	±	4.2	Hawthorne, honey, sweet
	Nonanal *	1103.98	76.8	±	39.2	fat, citrus, green
Esters	3-Methylbutyl acetate *	880.34	122.6	±	16.7	banana
	2-Methylbutyl acetate	882.62	13.5	±	23.4	fruit
	Ethyl hexanoate *	999.81	552.6	±	47.7	Pineapple
	Hexyl acetate *	1015.21	24.9	±	2.5	Fruit, herb
	Ethyl octanoate *	1198.16	3686.7	±	428.6	fruit, fat
	2-Phenylethyl acetate	1260.84	23.4	±	3.3	rose, honey, tobacco
	Ethyl decanoate *	1399.01	5706.5	±	629.4	grape
	Dibutyl hexanedioate	1772.74	43.9	±	7.6	
Hydrocarbons	2-Methyl heptane	743.57	875.2	±	902.3	
	3-Ethyl hexane	751.71	424.6	±	85.9	
	3-Ethyl octane	972.65	61.1	±	26.1	
	2,2,4,6,6-Pentamethyl heptane	989.05	7740.3	±	2245.9	
	2,2,4,4-Tetramethyl octane	1025.93	595.0	±	178.8	
	Dodecane, 2,6,10-trimethyl	1049.14	75.3	±	23.1	
Others	Acetic acid	692.27	2343.8	±	610.4	sour
	Methoxy-phenyl-oxime	928.85	1094.5	±	360.5	
	Eucalyptol	1029.41	15.8	±	27.5	

* The identification of the compound was carried out by the mass spectrum and LRI identical with a commercial standard compound. ^a^ Flavor descriptors from the Cornell University Flavornet (http://www.flavornet.org/flavornet.html (accesed on 28 September 2024)) and the Good Scents Company (http://www.thegoodscentscompany.com/index.html (accesed on 28 September 2024)).

## Data Availability

The authors declare that the data supporting the results of this work are accessible within the article. The data presented in this study are available on request from the corresponding author.
